# Pre-Analytical and Analytical Variables of Label-Independent Enrichment and Automated Detection of Circulating Tumor Cells in Cancer Patients

**DOI:** 10.3390/cancers12020442

**Published:** 2020-02-13

**Authors:** Claudia Koch, Simon A. Joosse, Svenja Schneegans, Okka J. W. Wilken, Melanie Janning, Desiree Loreth, Volkmar Müller, Katharina Prieske, Malgorzata Banys-Paluchowski, Ludwig J. Horst, Sonja Loges, Sven Peine, Harriet Wikman, Tobias M. Gorges, Klaus Pantel

**Affiliations:** 1 Department of Tumor Biology, Center of Experimental Medicine, University Medical Center Hamburg-Eppendorf, 20246 Hamburg, Germany; c.koch@uke.de (C.K.); s.joosse@uke.de (S.A.J.); s.schneegans@uke.de (S.S.); okka.wilken@stud.uni-goettingen.de (O.J.W.W.); m.janning@uke.de (M.J.); d.loreth@uke.de (D.L.); Ludwig-J.Horst@outlook.de (L.J.H.); sonjaloges@me.com (S.L.); h.wikman@uke.de (H.W.); t.gorges@uke.de (T.M.G.); 2 Department of Oncology, Hematology and Bone Marrow Transplantation with section Pneumology, Hubertus Wald Tumorzentrum, University Comprehensive Cancer Center Hamburg, University Medical Center Hamburg-Eppendorf, 20246 Hamburg, Germany; 3 Department of Gynecology and Gynecologic Oncology, University Medical Center Hamburg-Eppendorf, 20246 Hamburg, Germany; v.mueller@uke.de (V.M.); k.prieske@uke.de (K.P.); 4 Department of Gynecology, Asklepios Clinic Barmbek, 22307 Hamburg, Germany; maggiebanys@yahoo.de; 5 Department of Gynecology and Obstetrics, Marienkrankenhaus Hamburg, 22087 Hamburg, Germany; 6 Department of Transfusion Medicine, University Medical Center Hamburg-Eppendorf, 20246 Hamburg, Germany; s.peine@uke.de

**Keywords:** circulating tumor cells, CTC, enrichment, Parsortix, cancer metastasis

## Abstract

Circulating tumor cells (CTCs) are promising tools for risk prediction and the monitoring of response to therapy in cancer patients. Within the EU/IMI CANCER-ID consortium, we validated CTC enrichment systems for future inclusion into clinical trials. Due to the known heterogeneity of markers expressed on CTCs, we tested the Parsortix^®^ system (ANGLE plc) which enables label-independent CTC enrichment from whole blood based on increased size and deformability of these tumor cells compared to leukocytes. We performed extensive comparisons both with spiked-in blood models (i.e., MDA-MB-468 tumor cell line cells spiked at very low concentration into blood from healthy donors) and validated the protocol on actual clinical samples from breast, lung, and gastrointestinal cancer patients to define optimal conditions for CTC enrichment. Multiple parameters including cassette gap, separation pressure, and cell fixatives were compared in parallel. Also, the compatibility of blood collection tubes with whole genome amplification of isolated tumor cells was demonstrated and we furthermore established a workflow for semi-automated CTC detection using a quantitative cell imager. The established workflow will contribute to supporting the use of size-based CTC enrichment platforms in clinical trials testing the clinical validity and utility of CTCs for personalized medicine.

## 1. Introduction

Solid tumors are able to actively or passively shed tumor cells into the blood at times as early as primary tumor formation [1]. A subset of these circulating tumor cells (CTCs) harbors the potential to extravasate from the circulation and colonize distant organs. As 90% of cancer-related deaths are caused by distant metastasis, the presence and relevance of CTCs in the bloodstream has been intensively investigated over the past 10 years. CTCs have been shown to correlate significantly with the probability of metastatic relapse in various carcinomas [2]. These studies demonstrate that the capability of a tumor to release malignant cells into the blood presents a specific risk for the development of metastasis. In addition to CTC enumeration, the characterization of these rare cells holds great promise as they harbor valuable information on the patient’s current tumor profile and could therefore function as “liquid biopsy” [3,4].

The greatest challenge when detecting CTCs is their extremely low concentration in peripheral blood. A single CTC can be present in a background of approximately 10^8^–10^9^ blood cells, explaining why technical hurdles have limited the analysis of these cells in the past. Thus, to unlock the full potential of CTC research, extremely sensitive and specific enrichment methods have to be applied. Currently, more than 50 distinct CTC detection systems are available on the market or under development in different laboratories all over the world [5,6]. However, the FDA-cleared CellSearch^®^ system remains the “gold-standard” for capture of CTCs in a standardized and validated manner [7,8,9,10,11,12]. Due to its dependency on the epithelial cell adhesion molecule (EpCAM), this system is limited to EpCAM positive tumor cells with primarily epithelial phenotypes [13]. While this applies to most carcinoma cells, metastatic outspread is often associated with the transition of epithelial-like tumor cells into a more mesenchymal state (epithelial to mesenchymal transition, EMT), indicated by loss or downregulation of EpCAM expression [14,15]. Mesenchymal CTCs have been associated with greater invasiveness, chemo-resistance, and lower overall survival rates for patients [16,17,18]. Hence, the establishment of additional, non-EpCAM-based assays is of high importance.

Although new EpCAM-independent CTC technologies have already shown promising results [5], standardized protocols for most approaches are still lacking. The CANCER ID consortium (www.cancer-id.eu), funded by the European Innovative Medicine Initiative (IMI), aimed to close this gap and validate innovative CTC detection approaches for future clinical trials. This standardization of novel technologies and workflows is much needed to increase the acceptance of CTCs as biomarkers in the clinic.

The Parsortix^®^ system (ANGLE plc)-a size-based enrichment device - has been introduced to the CTC field and shown promising results across multiple tumor entities [19,20,21,22,23,24]. In addition to enumeration, the device has demonstrated its capabilities in isolating rare CTC clusters [24,25] and preserving cell viability following CTC enrichment [19,26]. When first introduced to the market, several individual research groups using this device applied different pre-analytical conditions (e.g., blood tubes for sample collection) as well as distinct separation protocols for CTC enrichment, making direct comparison of the Parsortix^®^ system performance across those research groups and studies very difficult.

Here, we conducted extensive spiking experiments to establish tumor cell capture rates using various pre-analytical and analytical conditions in spike-in model experiments and cross-validated the results by the analysis of peripheral blood samples from patients with different forms of cancer (breast, lung, and gastrointestinal). The complete workflow was tested for its compatibility with whole genome amplification (WGA) and molecular characterization of the amplified tumor cells. Finally, we additionally established a workflow for semi-automated CTC detection using the XCyto^®^ 10 quantitative cell imager. The established standardized workflows enable the enrichment of viable or fixed CTCs and open the avenue for precise enumeration and downstream analysis of patient-derived CTCs.

## 2. Materials and Methods

### 2.1. Cell Lines and Culture

The breast cancer derived cell lines MDA-MB-468 and MCF-7 acquired from ATCC (ATCC, Manassas, VA, USA), were chosen for analysis. Cells were cultured in cell culture flasks under standard conditions in humidified incubators at 37 °C with 10% CO_2_. Medium (Gibco-Life Technologies, Darmstadt, Germany) was employed as recommended by ATCC and fortified with 10% fetal bovine serum (Gibco-Life Technologies, Darmstadt, Germany, 1% L-glutamine (Gibco-Life Technologies, Darmstadt, Germany) and 1% penicillin/streptomycin (Gibco-Life Technologies, Darmstadt, Germany). Cell passaging was performed at 70% confluency. This study was approved by the local ethical review board under numbers PV3779 and PV5392.

### 2.2. Spiking of Healthy Donor Blood with Cell Line Cells

Cell line cells were prepared for spiking experiments by washing with 1 x PBS (Gibco-Life Technologies, Darmstadt, Germany) and incubating with 0.25% trypsin-EDTA (Gibco-Life Technologies, Darmstadt, Germany) for 5 min at 37 °C prior to resuspending in culture medium. The cell suspension was centrifuged at 190× *g* for 5 min after which the supernatant was discarded and the cells were resuspended in fresh culture medium. The cells were spread to a petri dish filled with medium, manually counted and picked under a light microscope. Defined cell counts of 50 MDA-MB-468 cells were added to blood samples from 55 healthy donors (HD). Subsequently, tubes containing ethylenediaminetetraacetic acid (EDTA) were used to collect 7.5 mL of whole blood from healthy volunteers. Following blood spiking, the Ficoll density-gradient-based Leucosep™ pre-enrichment (Greiner Bio One, Kremsmünster, Austria) was used to separate peripheral mononuclear cells (PBMCs) from whole blood according to the manufacturer’s instructions for selected spike experiments.

### 2.3. Blood Sample Collection and Processing

Patient blood samples were acquired in accordance to the World Medical Association Declaration of Helsinki and the guidelines for experimentation with humans by the Chambers of Physicians of the State of Hamburg (“Hamburger Ärztekammer”). All participants gave written informed consent prior to blood donation (Ethics Nr. PV3779 and PV5392). Blood was drawn directly into standard EDTA vacutainers, CellSave^®^ Preservative tubes (Menarini Silicon Biosystems, Florence, Italy), Circulating Tumour Cell TransFix/EDTA Vacuum Blood Collection Tubes (CTC-TVT tubes, CYTOMARK, Buckingham, UK), or Streck Cell-free DNA BCT tubes (Streck, La Vista, Nebraska, USA), respectively. EDTA blood was processed within 2 h of sample collection by the Parsortix^®^ system (ANGLE plc, Guildford, UK) and fixed blood within 24 h. In total, samples from 61 patients were analyzed in this study, including 48 metastatic breast cancer (mBC) as well as 6 metastatic non-small-cell lung cancer (mNSLC), 1 metastatic small-cell lung cancer (mSCLC), and 6 metastatic gastrointestinal (mGIC) cancer patients.

### 2.4. Tumor Cell Enrichment by the Parsortix^®^ System

The Parsortix^®^ system (ANGLE plc, Guildford, UK) is a benchtop microfluidic device designed for the size-based capture of rare cells from whole blood [19]. The blood sample is passed through an enclosed disposable cassette with a controlled liquid flow. The cassettes contain a stepped structure, gradually narrowing in diameter until reaching a final gap of 6.5 μm or 10 μm, respectively. The separation principle is based on the assumption that most tumor cells are larger in size and more rigid than normal, healthy blood cells [27]. These cells of interest are therefore retained by the critical gap of the separation cassette while all smaller cells continue to flow through the cassette and into a waste container. Following separation, the liquid flow through the cassette is reversed and the captured tumor cells are flushed out. Cells were directly harvested into cytospin funnels, centrifuged onto a glass slide (190× *g*, 3 min), dried overnight, and stored at −80 °C until further processing.

### 2.5. Immunocytochemistry

Tumor cells isolated with the Parsortix^®^ system were identified via immunocytochemistry. Dried cytospin slides were brought to room temperature (RT) and fixed with 2% PFA (Sigma Aldrich, Steinheim, Germany) for 10 min. The samples were washed with 0.5 mL of 1x-PBS prior to permeabilization with 0.1% Triton X 100/PBS (Sigma Aldrich, Steinheim, Germany) for 10 min. Following two additional wash steps, 10% AB-serum/PBS (BioRad, Rüdigheim, Germany) was applied for blocking (20 min). Directly Alexa Fluor 488 conjugated pan-keratin (AE1/AE3-eBioscience; C11-CellSignaling) and APC labelled CD45 (clone REA747-Miltenyi Biotec) antibodies were incubated for 60 min, followed by 5 min of DAPI-incubation (1 µg/mL). Subsequently, cytospins were covered with Prolong Gold Antifade Reagent (Thermo Fisher Scientific, Dreieich, Germany), sealed with a cover slip and examined by fluorescence microscopy (Axioplan2, Zeiss). Keratin-positive, DAPI-positive, CD45-negative cells with intact morphology were defined as tumor cells.

### 2.6. Measurement of Cell Size

MDA-MB-468 cell culture cells were trypsinized and resuspended in medium. The cell suspension was transferred to a glass slide via cytocentrifuge (190× *g*, 3 min). The resulting cytospins were left to dry overnight and stained with fluorescently labelled antibodies for pan-keratin and DAPI, as described above. Subsequently, 50 of the fluorescently labelled cells were photographed. The cell diameter was determined using the AxioVision LE64 microscope software (Zeiss) measurement tool and utilizing the borders of cytokeratin expression. An average was calculated from 50 separate cell measurements.

### 2.7. Whole Genome Amplification and DNA Quality Control

In order to test the feasibility of downstream genomic analyses, the complete workflow was tested for its compatibility with whole genome amplification (WGA) and molecular characterization of the genomic material. In brief, breast cancer cell line cells (MDA-MB-468) were transferred into EDTA or Transfix^®^ preserved healthy donor blood and processed by standard protocol (Parsortix^®^ system separation–99 mbar, cytospin and standard ICC). Subsequently, 10 single MDA-MB-468 and 5 background leukocytes were manually picked from the glass slides for each blood tube type and processed by WGA using the Ampli1™ WGA kit (Menarini Silicon Biosystems, Florence, Italy) according to the manufacturer’s instructions. 5 µg of gDNA were utilized as internal positive WGA control. Negative controls consisted of H_2_O. The quality of the amplified DNA was assessed by multiplex PCR producing 96, 108–166, 299, and 614 bp fragments from target sites in the *GAPDH* gene using the Ampli1™ QC Kit (Menarini Silicon Biosystems, Florence, Italy). To visualize PCR products, they were mixed with DNA Gel loading dye (6x) (Thermo Fisher Scientific, Dreieich, Germany) and applied to 1.2% agarose gels containing GelRed^®^ Nulceic Acid Gel Stain (Biotum, Fremont, CA, USA) at 1 µL per mL of agarose gel. The Quick-Load^®^ 100 bp DNA Ladder (New England Biolabs, Frankfurt am Main, Germany) was used as a size standard. PCR fragments were visualized using the Gene Genius bioimaging system (Syngene, Bangalore, India).

### 2.8. Image Analysis Using the XCyto 10 Platform

Immunocytochemistry-stained cytospins containing PBMCs (300 000), spiked with 1000 MCF-7 cells, were imaged using the XCyto^®^ 10 Quantitative Cell Imager (ChemoMetec, Lillerød Denmark). XCyto^®^ 10 is a high sensitivity image cytometer for suspended and adherent cells with a CCD 2.8 MP camera, four LEDs and 9 emission filters. A program was developed for semi-automatic detection of CTCs. First the slides were scanned with the XCyto^®^ 10 at 4× magnification. Acquisition times were 100 ms (milli seconds) for DAPI, and 500 ms for both Alexa Fluor 488 and APC fluorophores. The corresponding excitation light sources/emission filters were LED405/430-475 nm, LED488/513-555 nm, and LED635/665-676 nm, respectively. Within the XcytoView software, the “Xcyto 2-Sample 15-A v1” slide type was chosen for imaging as well as the 4× objective and the “Adherent, User defined–Fluorescence Mask” method for cell nucleus identification. The default analysis setup settings were applied except for a threshold intensity of 10,000 of the DAPI input channel, and a max area of 900 μm^2^.

The distinction of pan-keratin positive tumor cells against the background of CD45-positive PBMCs was established by plotting APC intensities of single cells against their corresponding intensity in the Alexa Fluor 488 channel. The data was visualized by application of bi-exponential scaling. Separation of both populations was achieved by gating the cells with high 488 nm signal and low 660 nm signal. The gating was set so that weakly positive cells were identified as well, and therefore some false positive cells were also included. Therefore, this pre-selection produced a gallery of potential CTCs which were then analyzed further by re-imaging at 20× magnification to manually exclude false positive hits. The same gating protocol was then reapplied to images of patient samples with identical staining and image acquisition parameters. The automated imaging set-up for CTC detection can be adjusted retrospectively, to further improve the gating parameters, making it an easily adaptable and time-saving tool for screening, e.g., large numbers of patient samples.

### 2.9. Statistics

Statistical analyses were performed using R version 3.6.1 (R Foundation for Statistical Computing) and In-Silico Online, version 2.1.2 [28]. Tumor cell recovery of spiking experiments of the three investigated fixed factors cassette gap (6.5 or 10 μm), separation pressure (23 or 99 mbar), and blood sample types (CellSave, EDTA, or Ficoll) were tested using a general linear model including interactions. Dependency of cassette gap (6.5 or 10 μm) on the recovery of CTCs in patient samples was tested by general linear modeling in Poisson distribution, using patient as within factor. McNemar’s exact test, Cohen’s kappa, and Kendall’s tau were employed to assess agreement between cassette gaps in CTC positive samples, as well as to assess agreement in detected CTCs using manual and automated (XCyto 10) screening. Difference between median cell counts was assessed by Wilcoxon signed rank test. A binomial test was employed to test which cassette gap outperformed the other.

## 3. Results

### 3.1. Tumor Cell Recovery of Different Cassette Gaps in Spiking Experiments

**Figure 1:**
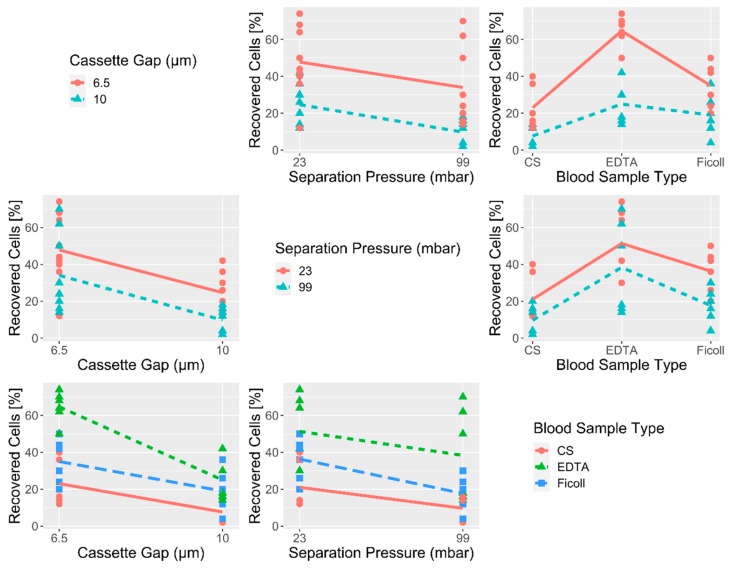
Visualization of initial spike experiment results. Correlation matrix showing the recovery of tumor cells under the different experimental conditions of the cassette gap (6.5 μm and 10 μm), separation pressure (23 mbar and 99 mbar), and the blood sample type (CellSave^®^-CS, EDTA and Ficoll).

Within the last years, ANGLE introduced a novel separation cassette with a decreased critical gap of 6.5 μm compared to the originally designed 10 μm. These cassettes could therefore theoretically allow the isolation of additional CTC populations with a smaller diameter. To test the performance of the 6.5 μm gap size cassettes in comparison to the original 10 μm gap size cassettes, we conducted extensive spiking experiments using the breast cancer cell line MDA-MB-468. MDA-MB-468 has a mean cell diameter of 13.5 μm (s = 2.03), with a measured range of 8.71–19.68 μm (Table S1). We focused on CTC capture rates for the different cassette gap sizes (6.5 μm and 10 μm), in 3 different blood sample types (EDTA, Ficoll, and CellSave^®^) and two separation pressures (23 mbar and 99 mbar) to allow a comprehensive overview (Table 1). Healthy donor (HD) blood collected in EDTA or CellSave^®^ tubes was spiked with a standard amount of 50 MDA-MB-468 cells. Samples drawn into EDTA were either directly processed or their PBMC fraction was pre-enriched via density gradient centrifugation (Ficoll) prior to separation with the Parsortix^®^ system. All conditions were tested in triplicate or more (*n* ≥ 3). Mean recoveries as well as standard deviations (s) are indicated in Table 1. Detailed results of the spike-in experiments are listed in Table S2. Overall, the cassettes with the 6.5 μm gap size led to a clear increase in the proportion of harvested tumor cells for each blood processing type and separation pressure (Table 1, Figure 1). This was confirmed in a generalized linear fixed-effects model (GLM) analysis showing that independent of protocol and blood sample type, a mean of 15.9% higher recovery was achieved with the cassettes with 6.5 μm gap size compared to 10 μm (Figure 1). Apart from recovery, a more efficient priming of 6.5 μm gap sized cassettes was observed, leading to an optimized blood flow within the separation cassette compared to the 10 μm version (Figure S1A). This increase in recovery comes at the cost of higher white blood cell (WBC) background (Figure S1B) and increased separation times (Table S2).

The 23 mbar (lower pressure) separation protocol allowed for higher tumor cell recovery than applying a greater separation pressure of 99 mbar (Figure 1), resulting in a mean increase of recovery of 10.8% for the lower pressure protocol, as calculated by GLM analysis. The most dramatic influence was detected when comparing different blood collection tubes. Here, EDTA outperformed Ficoll pre-enriched and CellSave^®^ fixed samples, showing an average 40.8% increase in recovery compared to CellSave^®^ fixed cells, independent of the cassette type and separation protocol used. Combining EDTA blood tubes with the 6.5 μm gap sized cassette further increases the mean recovery with an additional 24.3%, independent of the chosen protocol.

While the lower separation pressure (23 mbar) resulted in increased recoveries compared to the higher pressure (99 mbar) separation, it doubled the mean sample running time (Table S2). Therefore, although performing better, the 23 mbar protocol was abandoned for standard 7.5 mL whole-blood samples to allow for better sample turnover.

### 3.2. Analysis of Blood Samples from Breast Cancer Patients with Different Cassette Gaps

To confirm the increase of CTC capture with the 6.5 μm gap sized cassette in cancer patient samples, we analyzed 43 blood samples from 37 mBC patients progressing under their current therapy. 6 patients were monitored on a second visit. Disease progression in this cohort was clinically assessed by CT-scan or MRI. An overview of the clinical parameters of this patient cohort is given in Table S3. Matched 7.5 mL EDTA samples from each patient were processed with the 6.5 μm and the 10 μm gap sized cassettes. Overall, 32.4% (12/37 patients) of patients in our cohort were positive for ≥1 CTC and 13.5% (5/37 patients) for ≥5 CTCs when combining both measurements (6.5 μm and 10 μm runs) (Table S3). Overall positivity rates for ≥1 CTC remained comparably high at 32.6% when focusing on total blood samples analyzed (14/43 samples); however, the percentage of finding ≥5 CTCs increased to 20.9% (9/43 samples). A moderate agreement of CTC status (positive/negative) within samples between the two cassette gap sizes was assessed by Cohen’s kappa test (kappa = 0.57, 95% CI: (0.28, 0.86), *p* = 0.0018). Additionally, using a McNemar’s exact test, no detectable change in the CTC status (positive/negative) was determined by both cassettes within a sample (*p* = 0.13). Agreement between CTC counts across the complete sample cohort (37 patients, 43 samples) was moderate between the two cassette sizes (Kendall’s tau test: tau = 0.65, *p* = 2.4 × 10^−6^). However, the majority of measurements were CTC-negative for both samples (29/43 samples), which significantly influenced these results.

**Figure 2:**
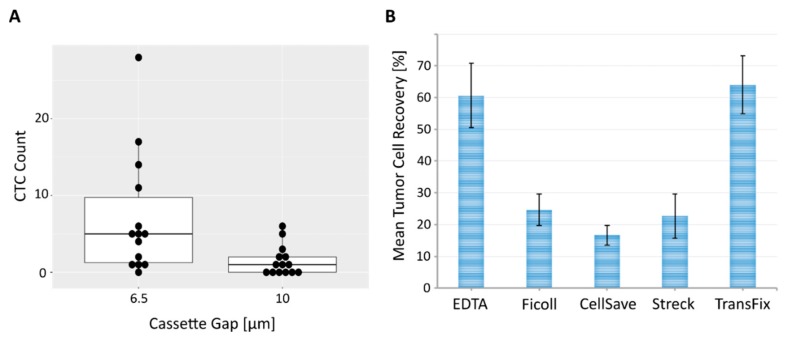
Performance comparison between 6.5 μm and 10 μm cassette gap as well as different blood sample types. (**A**) Box plot demonstrating distribution of CTC counts within the 14 CTC positive mBC patients detected in our cohort. Matched blood samples that showed ≥ 1CTC in either the 6.5 μm or 10 μm gap sized cassettes are depicted. Each dot represents a single patient. (**B**) Mean tumor cell recovery [%] calculated from spike experiments using MDA-MB-468 breast cancer cell line cells for different blood sample types. Cell line cells were spiked into healthy donor blood, processed via the Parsortix^®^ system (6.5 μm gap sized cassette, 99 mbar separation pressure) and subsequent cytospin and ICC staining. Depending on the sample type, blood was either processed directly (EDTA), immediately pre-enriched via density gradient centrifugation (Ficoll) or fixed for 24 h (CellSave^®^, Streck^®^, TransFix^®^) prior to separation with the Parsortix^®^ system. Recovery rates for each blood sample condition are indicated with standard deviation (black bars).

Looking more closely at the positive samples, 13/14 had ≥ 1 CTC detectable using the 6.5 μm gap sized cassette (92.9%) and 8/14 with the 10 μm gap sized cassette (57.1%). CTC counts with established prognostic relevance in mBC (≥5 CTCs) were detected in 7/14 samples (range: 1–28 CTCs) with the smaller separation gap (50%) compared to 2/14 samples (range: 1–6 CTCs) with the original, larger gap (14.3%). Only a weak agreement in CTC count was discerned within the positive samples between the two chip sizes (Kendall’s tau test: tau = 0.51, *p* = 0.023). Across all samples, a total of 99 CTCs were detected using the 6.5 μm versus a total of 21 CTCs with the 10 μm gap sized cassette, marking a 4.7-fold increase in CTC numbers using the smaller cassette diameter (Table S3). An exact binomial test performed on this data, resulted in a 92.9% probability of the smaller cassette gap recovering higher CTC counts than the larger cassette gap (95% CI: (0.70, 1.00), *p* = 0.0009). Finally, we performed a Wilcoxon signed rank test with continuity correction to test whether the median CTC count of the samples were equal with both cassette types. The median CTC counts between the 6.5 μm and 10 μm gap sized cassette differed (V = 102.5, *p* = 0.0018), as visualized in the corresponding box plots (Figure 2A). Conclusively, our data indicates that, while both cassette types show a moderate correlation in the CTC-positive and CTC-negative results they generate for each sample, the 6.5 μm gap sized cassette captures substantially higher CTC numbers in mBC patients, confirming its superiority for CTC analysis.

### 3.3. Effect of Blood Collection Tube on Tumor Cell Enrichment

Standard EDTA tubes are well suited when analyzing fresh samples that can be processed within 2–3 h following blood draw. However, clinical routine does not always allow such short processing time frames. Additionally, shipping of samples across sites for clinical studies requires some form of cell fixation to allow optimal sample processing. The CellSave^®^ preservation tube (CS) tested initially did not yield high recovery rates in combination with the Parsortix^®^ system (16.7%, s = 3.1, S99F separation protocol). Therefore, we extended our analysis to additional blood tubes, including Streck Cell-free DNA BCT tubes (Streck, La Vista, Nebraska, USA), which should technically allow the isolation of ctDNA and CTCs from the same tube [20], and Circulating Tumour Cell TransFix/EDTA Vacuum Blood Collection Tubes (CTC-TVT) by CYTOMARK (Buckingham, UK). Additional spiking experiments (50 MDA-MB-468 cells, S99F separation protocol, *n* = 3) resulted in a mean recovery of 21.5% (s = 6.2) for Streck^®^ tubes and 64% (s = 9.2) for TransFix^®^ stabilized blood (Table 2). Across all measured spike conditions, blood from EDTA and TransFix^®^ tubes therefore resulted in the best tumor cell recoveries (Figure 2B). We furthermore tested an additional separation protocol for EDTA samples using a medium separation pressure of 50 mbar (S50F). While this protocol did not significantly increase recovery (65.7%, s = 6.5) compared to the 99 mbar protocol (60.7%, s = 10.1), it did improve the morphological intactness of unfixed tumor cells as well as of the leukocyte background. Detailed results for all blood tube types are listed in Table S2.

### 3.4. Validation of Optimized Protocol on Blood Samples from Cancer Patients

To allow for a direct comparison of all blood tube types analyzed in prior spike experiments (EDTA, CellSave^®^, Streck^®^, and TransFix^®^), and to compare the 99 mbar and 50 mbar EDTA separation protocols in clinical samples, blood was drawn in parallel from 6 metastatic non-small-cell lung cancer, 1 metastatic small-cell lung cancer, and 6 metastatic gastrointestinal cancer patients into all blood tube types and analyzed via the Parsortix^®^ system. Clinical data for this patient collective is detailed in Table S4.

**Figure 3:**
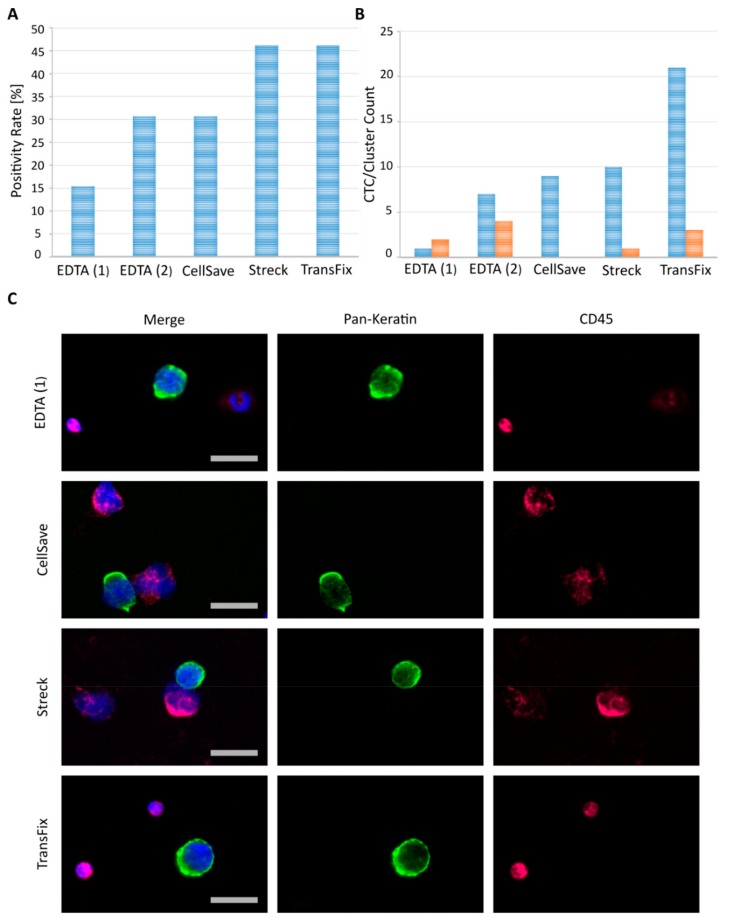
Comparison of blood collection tubes in clinical patient samples drawn and processed in parallel. EDTA (1) indicates samples were processed by the 99 mbar separation protocol, while EDTA (2) samples were run using the reduced 50 mbar Parsortix^®^ protocol. Fixed samples were stored for 24 h following blood draw and separated using the high pressure Parsortix^®^ protocol (99 mbar). All samples were subsequently cytospun and stained by fluorescent antibodies against and DAPI (blue). (**A**) Percentage of CTC-positive patients (defined as ≥ 1 CTC per 7.5 mL of blood) across the 14 cancer patients tested. (**B**) Blue bars: Overall single CTC count detected in the 14 cancer patients tested in parallel for each collection tube. Orange bars: Amount of CTC clusters (≥2 directly attached CTCs) detected in the 14 cancer patients tested in parallel for each collection tube. (**C**) Representative CTC and leukocyte images from selected index patients within our cohort. Images were taken manually with a standard fluorescence microscope at 40× magnification. Size bars equal 20 μm.

The highest rates of finding ≥1 CTC in patients were reached at 46.2% (6/13 patients) for blood collected into the Streck^®^ and TransFix^®^ tubes (Figure 3A). This was followed by CellSave^®^ fixed and EDTA blood (processed by the 50 mbar separation protocol) at 30.8% CTC-positivity (4/13 patients). Unfixed EDTA samples processed by a high separation pressure of 99 mbar rendered the lowest CTC positivity rates with 15.4% (2 out of 13 patients). While the overall percentage of CTC-positive patient cases were equal for Streck^®^ and TransFix^®^ fixed, as well as for CellSave^®^ fixed and EDTA blood (50 mbar separation), the total amount of CTCs detected via immunocytochemistry (ICC) was considerably higher in TransFix^®^ samples (Figure 3B, Table S5). Also, EDTA (50 mbar separation) and TransFix^®^ samples resulted in the highest total amount of CTC clusters compared to the other tube types and separation protocols (Figure 3B, Table S5). CTC clusters were defined as ≥2 CTCs closely attached to one another. Representative ICC staining images of tumor cells detected using the different blood tubes and separation protocols are depicted in Figure 3C. Cell morphology of tumor cells and leucocytes remained intact following enrichment and analysis with our protocols. However, leucocyte nuclei appeared slightly enlarged or “puffy” when blood was treated with CellSave^®^ or Streck^®^ fixatives (Figure 3C). This effect was not detected in EDTA or TransFix^®^ blood samples (Figure 3C). Additionally, staining and therefore fluorescence signal intensity varied across tube types, especially for the leukocyte background. Heterogeneous staining intensity of leukocytes is demonstrated for an EDTA and Streck fixed patient sample in Figure 3C. Overall best and most homogeneous ICC staining performance was achieved using TransFix^®^ preserved blood (Figure 3C).

In conclusion, TransFix^®^ preservation tubes proved optimal in combination with Parsortix^®^ separation and subsequent ICC staining on cytospins, resulting in high overall positivity rates of 46.2% (Figure 3A) and highest total CTC counts detected (Figure 3B). Furthermore, these tubes guarantee intact tumor cell morphology (Figure 3C) and enable detection of CTC clusters (Table S5).

### 3.5. Downstream Molecular Analysis of Single Tumor Cells Following Enrichment

Molecular analysis on a genomic level represents a key tool allowing further in-depth characterization of CTCs following their successful detection. While a multitude of different methods are available and routinely applied (e.g., next generation sequencing, ddPCR, microarrays), in most cases, pre-amplification of genomic material is necessary to enable conclusive and robust results. This is especially true when focusing on single tumor cells with an initial DNA content of a few picograms. The most prevalent method of increasing DNA quantity for subsequent molecular analysis is currently whole genome amplification (WGA).

To test the feasibility and efficiency of successful WGA following Parsortix^®^ separation and subsequent ICC staining on cytospins, additional spiking experiments were conducted. Because patient derived CTCs can be genetically heterogeneous [29], we spiked blood from healthy donors with MDA-MB-468 tumor cell line cells, fixed with either Transfix^®^ preservative or in standard EDTA tubes to have identical starting material in all experiments and control for other confounding factors. Next, cell separation was performed using the Parsortix^®^ system (99 mbar protocol). Following ICC staining, 10 single tumor cell line cells and 5 leukocytes were picked from glass slides by micromanipulation and processed by WGA (Ampli1™ WGA kit, Menarini Silicon Biosystems). Amplified cells were subsequently subjected to DNA quality control (Ampli1™ QC kit, Menarini Silicon Biosystems) and analyzed via gel electrophoresis. High DNA integrity was defined as ≥3 GAPDH amplification products detected in quality control, while medium DNA integrity was defined as ≥2 GAPDH amplification products. WGA was successful in generating high DNA integrity for 80% (12/15 single cells, ≥3 GAPDH amplification products) of the single cells picked from EDTA as well as from TransFix^®^ preserved blood (Figure S2). For TransFix^®^, an additional medium quality sample was generated (1/15 single cells; ≥2 GAPDH amplification products), leading to an overall success rate of 87% (13/15 single cells). In total, highest achievable DNA quality (4/4 GAPDH amplification products) was seen for 73% (11/15 single cells) of single cells picked from TransFix^®^ and 46% (7 out of 15 single cells) of single cells from EDTA blood (Figure S2).

In conclusion these results indicate that both EDTA and TransFix^®^ blood tubes are suitable not only for enrichment of CTCs via the Parsortix^®^ system, but also allow for the robust amplification of single cell DNA at excellent quality for subsequent molecular analysis.

### 3.6. Compatibility with an Automated CTC Screening Device

**Figure 4:**
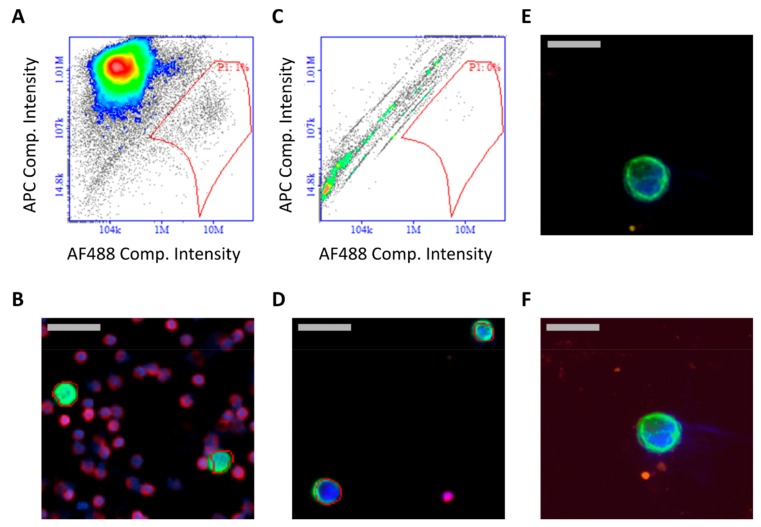
Semi-automated CTC evaluation using the XCyto^®^ 10 imaging system. (**A**) Image of the plot used for setting up a suitable gating to distinguish tumor cells from PBMCs. APC intensities (CD45 on leukocytes) of individual cells are plotted against their intensities in the Alexa Fluor 488 (pan-keratin on MCF-7 cells) channel. Cells with low CD45 (APC) and high pan-keratin (Alexa Fluor 488) signals were considered as tumor cells. Gate P1 was chosen so that a maximal separation of tumor cells from PBMCs was achieved. (**B**) Overlay of the P1 gate with a section of the original image. Cell nuclei were visualized with DAPI (blue), tumor cell marker pan-keratin- Alexa Fluor 488 was used (green) and CD45-APC (red) as a negative selection marker for PBMCs. Grey scale bar represents 40 μm. (**C**) Exemplary image of the gating from a patient sample. Cells with low APC- and high Alexa Fluor 488 signals were considered as potential CTCs in gate P1. (**D**) Overlay of positive hits of the P1 gate with a section from the original image. Grey scale bar represents 40 μm. (**E**) CTC from patient sample no. 13 imaged with 2 different settings. Above: CTC imaged with 40× objective of Axioplan2 (Zeiss) microscope. Grey scale bar represents 20 μm. (**F**) The same cell was imaged with XCyto^®^ 10 (20× objective). Grey scale bar represents 20 μm.

Manual evaluation of CTC counts remains user-dependent and time-consuming. To further improve the comparability of results generated with the Parsortix^®^ protocols defined in this study, we additionally established a workflow for semi-automated CTC detection using the XCyto^®^ 10 quantitative cell imager (ChemoMetec). For this, a gating protocol was established using blood from healthy donors spiked with MCF-7 tumor cell line cells to distinguish tumor cells (pan-keratin^high^/CD45^low^) and surrounding leukocyte background (pan-keratin^low^/CD45^high^) according to their respective Alexa Fluor 488 and APC signal intensities (Figure 4A,B). The same gating was then applied on patient samples processed through the Parsortix^®^ device. Cell counts identified by this gating were subsequently compared with the results of manual screening by fluorescence microscope.

In total, a set of 27 blood samples originating from 16 mBC patients was evaluated (Figure 4C,D). Samples of the analyzed cohort consisted of blood collected for the prior comparison of 6.5 μm and 10 μm cassette gaps (Table S3), as well as 18 additional blood samples from 10 mBC patients (mBC_38 to mBC_48) collected and processed solely by the 6.5 μm cassette gap (clinical data in Table S3). Both microscopes had comparable results with respect to image resolution and signal intensities in the different fluorescence channels (Figure 4E,F). 48.1% (13/27) of patient samples were found to be negative for CTCs using both screening approaches (Table S6). By manual screening, 44.4% (12/27) samples were found to be CTC-positive (Table 3). With the exception of two additional patient samples, these same samples were also identified to harbor CTCs after imaging with the XCyto^®^ 10 (48.1%, 14/27). Of these 14 CTC-positive patient samples, 97 CTCs were identified in total by using the automated settings, in comparison to 98 CTCs from manual screening (Table 3). For the majority of samples (74.1%, 20/27) equal CTC levels were detected with both screening approaches (Table 3 and Table S6). This was underlined by the result of a Wilcoxon signed rank test with continuity correction, indicating that the median CTC count of the samples were equal with both evaluation approaches (V = 14.5, *p* = 1). Furthermore, a very strong agreement in CTC count was discerned within the total 27 samples between the two screening methods (Kendall’s tau test: tau = 0.917, *p* = 2.22 × 10^−16^). When focusing on CTC-positive cases only, manual screening outperformed semi-automated screening in 21.4% (3/14) of samples analyzed. Vice-versa, in 28.6% (4/14) of CTC-positive samples analyzed, semi-automated screening outperformed manual screening (Table 3). However, differences were mostly confined to a small number of cells (range Δ: 1–4 cells). In line with this, a very strong agreement in CTC count was observable within the CTC-positive samples between the two screening methods (Kendall’s tau test: tau = 0.902, *p* = 3.79 × 10^−5^).

## 4. Discussion

The Parsortix^®^ system is a flexible platform which can allow some user-defined modification of various parameters in addition to cassette gap size (6.5 μm and 10 μm), including blood sample type and separation pressures. This allows for a versatile applicability of the device while in parallel complicating the determination of standardized protocols. In depth systematic comparisons are therefore warranted in order to ensure optimal device performance. In this study, we extensively evaluated a multitude of different parameters influencing the tumor cell capture efficiency of the Parsortix^®^ system and established optimized enrichment protocols ensuring robust performance.

Beginning with extensive spiking experiments mimicking clinical blood samples, we could show that the 6.5 μm cassette gap dramatically improves the amount of captured tumor cells in comparison to the 10 μm cassette gap (Table 1, Figure 1). The breast cancer cell line used for these initial experiments (MDA-MB-468) displayed an average cell diameter of 13.5 μm (s = 2.03), with a measured range of 8.71–19.68 μm (Table S1). Its diameter therefore lends itself to enrichment with both cassette gap sizes while its size distribution mirrors the morphological heterogeneity commonly seen between tumor cells [30,31]. In EDTA samples, which showed the highest overall mean recovery rates in these initial spiking experiments, an increase from 34% (s = 6.9) to 68.7% (s = 5.0) of mean recovery was demonstrated when using the 6.5 μm cassette gap instead of the 10 μm gap (Table 1). The results of our spiking experiments were validated on 43 blood samples of a cohort of 37 mBCa patients. Analyzing parallel blood draws via the Parsortix^®^ system, subsequent cytospin and ICC staining corroborated the superiority of the 6.5 μm cassette gap (Figure 2A), indicating a significant improvement compared to the 10 μm cassette gap used in prior studies [19,20]. We would therefore recommend the 6.5 μm cassette gap size for future Parsortix^®^ system studies.

Drawing blood into standard EDTA vacutainers is cost efficient and well suited for direct processing of fresh clinical samples within a few hours. However, this is not practical in clinical settings of large multi-center trials which usually require shipments over 24–96 h from the clinical site to the central laboratory. Therefore, we additionally tested various commercially available blood fixatives for their tumor cell recovery performance in combination with the Parsortix^®^ system. In spike experiments, TransFix^®^ preservation tubes showed overall best mean recovery rates, similarly high to EDTA spiked blood samples, while CellSave^®^ and Streck^®^ fixed blood performed less well (Table 2, Figure 2B). TransFix^®^ preservation tubes are designed to stabilize blood for up to 5 days, offering a wide window for sample processing. In clinical samples drawn and analyzed in parallel for the different sample types, combining TransFix^®^ preserved blood with Parsortix^®^ enrichment (6.5 μm, 99 mbar) resulted in acceptable CTC positivity rates of 46.2% across patients and the highest overall CTC count (Table S5, Figure 3A,B). The fixatives outperformed EDTA blood in clinical samples when looking at total single CTC counts (Figure 3B). This discrepancy to the results of our spike experiments could be rooted in higher fragility of patient CTCs compared to cultured tumor cell line cells. In contrast to cell lines, CTCs are influenced by a multitude of adverse factors (e.g., therapy, disease stage, blood passage etc.) which could lead to the observed benefit when using stabilizing reagents. However, the EDTA and lower pressure separation (50 mbar) protocol did result in the highest amount of CTC clusters detected in clinical samples (Table S5, Figure 3B), thereby shifting the reasoning somewhat in favor of an EDTA-based approach compared to fixatives such as CellSave^®^. Both EDTA and TransFix^®^ samples delivered intact, high quality cells, as demonstrated by quality control of whole genome amplification performed on single cell basis (Figure S2). In conclusion, we would suggest one of two protocols, depending on the research question posed as well as the logistics of the study in question. Fresh EDTA blood and 50 mbar separation represent the best option when cell viability is of highest importance (e.g., for subsequent attempts at cell culture or RNA analysis [19,32]). For morphological characterizations using ICC and subsequent molecular analysis based on DNA, samples preserved in TransFix^®^ (99 mbar separation) lead to the highest CTC yield and offer the possibility of prolonged sample shipment and extended processing windows.

A common point of criticism for novel CTC enrichment platforms is the necessity of manual instead of automated tumor cell calling. This manual sample evaluation is time consuming and limits comparability across different laboratory sites and clinical centers. Additionally, defining standardized staining protocols and evaluation criteria is necessary to allow for direct CTC comparison across sites. To address these issues, the feasibility of combining a new semi-automated CTC screening device, the XCyto^®^ 10 quantitative cell imager, with Parsortix^®^-based CTC enrichment was evaluated in this study. The high concordance of CTC-positive patients and the total number of identified CTCs between both semi-automated XCyto^®^ 10 based and traditional manual identification demonstrated the suitability of the XCyto^®^ 10 imaging platform for CTC screening purposes (Table 3). Automated sample scanning time using the XCyto^®^ 10 was faster (appr. 10 min per slide) compared to manual scanning (appr. 20 min per slide), resulting in an image gallery of potential CTCs (“hits”) displayed for the user in a similar manner to the CellSearch^®^. Being able to reapply a standardized set of gating parameters to a whole set of patients within a study cohort has the potential to increase reproducibility and furthermore to reduce bias from individual image analysis.

While the workflow resulting from this study stems from extensive experiments, some limitations require mentioning. The main limitation is that switching to the 6.5 μm gap sized cassette increased processing time and white blood cell background (Table S2, Figure S1). Sample processing time can be managed by using a high separation pressure of 99 mbar. Average processing times of 2 h for a complete EDTA (50 mbar) and 1.5 h for a TransFix sample (99 mbar) were deemed acceptable for sufficient sample turnover. White blood cell background has been shown to lie at 200–800 nucleated cells per mL of processed blood when using the 6.5 μm gap sized cassette [33]. This is known to be donor dependent [20], yet translates to an at least 10^5^ fold depletion of white blood cells [33]. In combination with our approach to CTC visualization, comprised of cytospin and subsequent ICC staining, this background leukocyte level is of no hindrance. Single CTCs detected on the cytospin can easily be picked via micromanipulation for subsequent molecular characterization. Additionally, this approach allows for sample storage and does not necessitate direct staining and evaluation (such as in cassette enumeration would), thereby circumventing long processing times and increasing comparability between samples. Another point of criticism might be the limited patient collective processed in this study as well as the mixed tumor entities of our cohort. This point is somewhat compensated by the fact that the entities tested (mNSCLC and mGIC) are known to be difficult for CTC enrichment [13,34,35]. A positivity rate of 46.2% in these patients therefore speaks to the value of our established workflow. Additionally, establishing protocols that demonstrate superiority across multiple tumor entities increase credibility and translational value of the tested method.

This study represents an extensive attempt at optimizing the performance for size-based CTC enrichment via the Parsortix^®^ system. How well the workflow established in this study performs across additional tumor entities will need to be further evaluated in large clinical studies, similar to those performed for the FDA-cleared CellSearch^®^ system [7,8,9,36,37,38]. However, we believe that the protocols established here represent a valuable first step towards methodological standardization of this promising CTC enrichment platform and show a clear path for inclusion into future clinical trials.

## 5. Conclusions

Extensive examinations of pre-analytical and analytical variables using the label-independent Parsortix^®^ system were performed in this study. Initial experimental results with spiked tumor cell line cells were cross-validated with clinical cancer patient samples and resulted in the identification of robust workflows for CTC detection utilizing this enrichment platform.

Depending on the nature of further downstream analysis, a combination of either EDTA blood samples with medium separation pressure or TransFix^®^ preservative tubes with high separation pressure lead to optimal CTC detection from whole blood. Intact tumor cell morphology was maintained, as confirmed via immunocytochemical staining for both workflows. Furthermore, whole genome amplification on a single cell basis, a prerequisite for multiple downstream analyses, was highly efficient. Therefore, the established standardized workflows enable the enrichment of viable or fixed CTCs and allow for precise enumeration and successful downstream analysis of patient-derived CTCs.

## Figures and Tables

**Table 1:** Mean tumor cell recovery rates determined from spike experiments using MDA-MB-468 tumor cell line cells. Recovery percentages and standard deviation (s) for each cassette type, blood processing type and separation pressure are indicated.

Cassette Gap	Protocol	Blood Processing and Tube Type					
		EDTA		Ficoll		CellSave	
		Recovery [%]	s [%]	Recovery [%]	s [%]	Recovery [%]	s [%]
10 μm	23 mbar	34	6.9	27.3	8.1	12.7	1.2
	99 mbar	16	2.0	10.7	6.1	2.7	1.2
6.5 μm	23 mbar	68.7	5.0	45.3	4.2	29.3	15.1
	99 mbar	60.7	10.1	24.7	5.0	16.7	3.1

**Table 2:** Mean tumor cell recovery rates determined from spike experiments (using MDA-MB-468 tumor cell line cells) for 6.5 μm cassette gap and various additional blood sample fixatives. Recovery percentages and standard deviation (s) for each blood processing type and tube are indicated.

Cassette Gap	Protocol	Mean Recovery [%]				
		EDTA	Ficoll	CellSave	Streck	TransFix
6.5 μm	99 mbar	60.7	24.7	16.7	22.7	64.0
	s [%]	10.1	5.0	3.1	7.0	9.2

**Table 3:** Summary of CTC-positive mBC patient samples and their corresponding CTC counts analyzed by either manual or XCyto^®^ 10 -based semi-automated screening. Cases in which one approach yielded higher CTC counts are marked in bold. The evaluation contains CTC-positive samples from prior analysis (Table S3) as well as 6 additional CTC-positive mBC samples (Table S3, Sheet 2). In the cases of “re-evaluated” samples (1) indicates samples processed via the 6.5 μm cassette gap and (2) samples that were separated via the 10 μm gap size. The 6 additional blood samples were solely processed using the narrow 6.5 μm cassette gap. All samples were drawn in EDTA.

Sample No. (CTC pos.)	Sample ID	CTC Count (Manual Screening)	CTC Count (XCyto10 Screening)
1	mBCa_15 (1)	14	13
2	mBCa_15 (2)	6	6
3	mBCa_22 (1)	1	2
4	mBCa_22 (2)	0	1
5	mBCa_26 (1)	5	5
6	mBCa_27 (1)	4	4
7	mBCa_27 (2)	0	1
8	mBCA_29 (1)	14	17
9	mBC_38	2	2
10	mBC_39	1	1
11	mBC_40	42	38
12	mBC_41	1	1
13	mBC_42	7	5
14	mBC_43	1	1
Total CTC count detected		98	97

## Supplementary Materials

The following are available online at https://www.mdpi.com/2072-6694/12/2/442/s1, Figure S1: Cassette priming and white blood cell (WBC) count, Figure S2: Efficiency of whole genome amplification (WGA) and results of DNA quality control on single cells processed with our optimized protocols. MDA-MB-468 cells were spiked into EDTA or TransFix^®^ blood and processed via Parsortix^®^ (6.5 μm cassette, 99 mbar). Subsequently, cells were stained, manually picked and DNA amplified using the Ampli1™ WGA kit (Menarini Silicon Biosystems). 10 single tumor cell line cells (1–10) and 5 leukocytes (L1–L5) were processed per blood tube type. gDNA was applied as positive (+) and H_2_O as negative (−) control. Following amplification, DNA was subjected to quality control using the Ampli1™ QC Kit (Menarini Silicon Biosystems) and visualized via agarose gel electrophoresis, Table S1: Cell diameters measured for MDA-MB-468 breast cancer cell line cells. Average and standard deviation was assessed from 50 separate cell measurements, Table S2: Detailed recovery results using MDA-MB-468 cells spiked into different blood sample types and processed with various experimental Parsortix™ conditions. Each measurement was carried out in triplicates and absolute as well as averaged values (including s = standard deviation) are indicated. Additionally, average processing time is noted, Table S3: Information on mBC patients providing blood samples for the Parsortix™ cassette gap comparison and the testing of semi-automated evaluation using the XCyto 10 platform. (Sheet1) Absolute CTC counts detected for the 37 mBC patients enrolled in this study are indicated. Each patient donated matched EDTA blood samples at blood draw to allow for direct comparison of 6.5 μm and 10 μm cassette gap performance. A second sample set was collected for 6 mBCa patients. (Sheet2) Basic clinical patient data available for our analyzed mBC cohort including additional patients for comparison of manual vs. semi-automated sample screening. Abbreviations include: pos–positive, neg–negative, and nA–not available, Table S4: Clinical information on the mixed cancer patient collective analyzed for blood tube comparison (n = 13). Clinical samples were obtained from 6 metastatic non-small-cell lung cancer (mNSCLC), 1 metastatic small-cell lung cancer (mSCLC) and 6 metastatic gastrointestinal cancer (mGIC) patients, Table S5: Detailed recovery results for 13 clinical cancer patient samples drawn in parallel into different blood tubes and processed with the 6.5 μm cassette gap via the Parsortix™ system. Clinical samples were obtained from metastatic non-small-cell lung cancer (mNSCLC), small-cell lung cancer (SCLC) and metastatic gastrointestinal cancer (mGIC) patients. In cases in which CTCs were found, results are presented as (x/y), x = total number of single CTCs, y = total number of CTC clusters detected, Table S6: Number of CTCs in blood samples from metastatic breast cancer patients detected by manual screening and by the automated XCyto 10 platform.

## References

[1] Kang Y., Pantel K. (2013). Tumor cell dissemination: emerging biological insights from animal models and cancer patients. Cancer Cell.

[2] Alix-Panabieres C., Pantel K. (2016). Clinical Applications of Circulating Tumor Cells and Circulating Tumor DNA as Liquid Biopsy. Cancer Discov..

[3] Pantel K., Alix-Panabieres C. (2019). Liquid biopsy and minimal residual disease - latest advances and implications for cure. Nat. Rev. Clin. Oncol..

[4] Keller L., Pantel K. (2019). Unravelling tumour heterogeneity by single-cell profiling of circulating tumour cells. Nat. Rev. Cancer.

[5] Alix-Panabieres C., Pantel K. (2014). Challenges in circulating tumour cell research. Nat. Rev. Cancer.

[6] Joosse S.A., Gorges T.M., Pantel K. (2015). Biology, detection, and clinical implications of circulating tumor cells. EMBO Mol. Med..

[7] Cohen S.J., Punt C.J., Iannotti N., Saidman B.H., Sabbath K.D., Gabrail N.Y., Picus J., Morse M., Mitchell E., Miller M.C. (2008). Relationship of circulating tumor cells to tumor response, progression-free survival, and overall survival in patients with metastatic colorectal cancer. J. Clin. Oncol..

[8] Cristofanilli M., Budd G.T., Ellis M.J., Stopeck A., Matera J., Miller M.C., Reuben J.M., Doyle G.V., Allard W.J., Terstappen L.W. (2004). Circulating tumor cells, disease progression, and survival in metastatic breast cancer. N. Engl. J. Med..

[9] De Bono J.S., Scher H.I., Montgomery R.B., Parker C., Miller M.C., Tissing H., Doyle G.V., Terstappen L.W., Pienta K.J., Raghavan D. (2008). Circulating tumor cells predict survival benefit from treatment in metastatic castration-resistant prostate cancer. Clin. Cancer Res..

[10] Riethdorf S., Fritsche H., Muller V., Rau T., Schindlbeck C., Rack B., Janni W., Coith C., Beck K., Janicke F. (2007). Detection of circulating tumor cells in peripheral blood of patients with metastatic breast cancer: a validation study of the CellSearch system. Clin. Cancer Res..

[11] Miller M.C., Doyle G.V., Terstappen L.W. (2010). Significance of Circulating Tumor Cells Detected by the CellSearch System in Patients with Metastatic Breast Colorectal and Prostate Cancer. J. Oncol..

[12] Scher H.I., Jia X.Y., de Bono J.S., Fleisher M., Pienta K.J., Raghavan D., Heller G. (2009). Circulating tumour cells as prognostic markers in progressive, castration-resistant prostate cancer: a reanalysis of IMMC38 trial data. Lancet Oncol..

[13] Riethdorf S., O’Flaherty L., Hille C., Pantel K. (2018). Clinical applications of the CellSearch platform in cancer patients. Adv. Drug Deliv. Rev..

[14] Tam W.L., Weinberg R.A. (2013). The epigenetics of epithelial-mesenchymal plasticity in cancer. Nat. Med..

[15] Gorges T.M., Tinhofer I., Drosch M., Rose L., Zollner T.M., Krahn T., von Ahsen O. (2012). Circulating tumour cells escape from EpCAM-based detection due to epithelial-to-mesenchymal transition. BMC Cancer.

[16] Yu M., Bardia A., Wittner B.S., Stott S.L., Smas M.E., Ting D.T., Isakoff S.J., Ciciliano J.C., Wells M.N., Shah A.M. (2013). Circulating breast tumor cells exhibit dynamic changes in epithelial and mesenchymal composition. Science.

[17] Yokobori T., Iinuma H., Shimamura T., Imoto S., Sugimachi K., Ishii H., Iwatsuki M., Ota D., Ohkuma M., Iwaya T. (2013). Plastin3 Is a Novel Marker for Circulating Tumor Cells Undergoing the Epithelial-Mesenchymal Transition and Is Associated with Colorectal Cancer Prognosis. Cancer Res..

[18] Mego M., Mani S.A., Lee B.N., Li C., Evans K.W., Cohen E.N., Gao H., Jackson S.A., Giordano A., Hortobagyi G.N. (2012). Expression of epithelial-mesenchymal transition-inducing transcription factors in primary breast cancer: The effect of neoadjuvant therapy. Int. J. Cancer.

[19] Hvichia G.E., Parveen Z., Wagner C., Janning M., Quidde J., Stein A., Muller V., Loges S., Neves R.P., Stoecklein N.H. (2016). A novel microfluidic platform for size and deformability based separation and the subsequent molecular characterization of viable circulating tumor cells. Int. J. Cancer.

[20] Chudziak J., Burt D.J., Mohan S., Rothwell D.G., Mesquita B., Antonello J., Dalby S., Ayub M., Priest L., Carter L. (2016). Clinical evaluation of a novel microfluidic device for epitope-independent enrichment of circulating tumour cells in patients with small cell lung cancer. Analyst.

[21] Xu L., Mao X., Imrali A., Syed F., Mutsvangwa K., Berney D., Cathcart P., Hines J., Shamash J., Lu Y.J. (2015). Optimization and Evaluation of a Novel Size Based Circulating Tumor Cell Isolation System. PLoS ONE.

[22] Gorges K., Wiltfang L., Gorges T.M., Sartori A., Hildebrandt L., Keller L., Volkmer B., Peine S., Babayan A., Moll I. (2019). Intra-Patient Heterogeneity of Circulating Tumor Cells and Circulating Tumor DNA in Blood of Melanoma Patients. Cancers (Basel).

[23] El-Heliebi A., Hille C., Laxman N., Svedlund J., Haudum C., Ercan E., Kroneis T., Chen S., Smolle M., Rossmann C. (2018). In Situ Detection and Quantification of AR-V7, AR-FL, PSA, and KRAS Point Mutations in Circulating Tumor Cells. Clin. Chem..

[24] Janning M., Kobus F., Babayan A., Wikman H., Velthaus J.L., Bergmann S., Schatz S., Falk M., Berger L.A., Bottcher L.M. (2019). Determination of PD-L1 Expression in Circulating Tumor Cells of NSCLC Patients and Correlation with Response to PD-1/PD-L1 Inhibitors. Cancers (Basel).

[25] Gkountela S., Castro-Giner F., Szczerba B.M., Vetter M., Landin J., Scherrer R., Krol I., Scheidmann M.C., Beisel C., Stirnimann C.U. (2019). Circulating Tumor Cell Clustering Shapes DNA Methylation to Enable Metastasis Seeding. Cell.

[26] Franken A., Driemel C., Behrens B., Meier-Stiegen F., Endris V., Stenzinger A., Niederacher D., Fischer J.C., Stoecklein N.H., Ruckhaeberle E. (2019). Label-Free Enrichment and Molecular Characterization of Viable Circulating Tumor Cells from Diagnostic Leukapheresis Products. Clin. Chem..

[27] Chen L., Bode A.M., Dong Z. (2017). Circulating Tumor Cells: Moving Biological Insights into Detection. Theranostics.

[28] Joosse S.A. In-Silico Online (version 2.1.2). http://in-silico.online.

[29] Joosse S.A., Pantel K. (2016). Genetic traits for hematogeneous tumor cell dissemination in cancer patients. Cancer Metastasis Rev..

[30] Park S., Ang R.R., Duffy S.P., Bazov J., Chi K.N., Black P.C., Ma H. (2014). Morphological differences between circulating tumor cells from prostate cancer patients and cultured prostate cancer cells. PLoS ONE.

[31] Follain G., Osmani N., Azevedo A.S., Allio G., Mercier L., Karreman M.A., Solecki G., Garcia Leon M.J., Lefebvre O., Fekonja N. (2018). Hemodynamic Forces Tune the Arrest, Adhesion, and Extravasation of Circulating Tumor Cells. Dev. Cell.

[32] Gorges T.M., Kuske A., Rock K., Mauermann O., Muller V., Peine S., Verpoort K., Novosadova V., Kubista M., Riethdorf S. (2016). Accession of Tumor Heterogeneity by Multiplex Transcriptome Profiling of Single Circulating Tumor Cells. Clin. Chem..

[33] Miller M.C., Robinson P.S., Wagner C., O’Shannessy D.J. (2018). The Parsortix Cell Separation System-A versatile liquid biopsy platform. Cytometry A.

[34] O’Flaherty L., Wikman H., Pantel K. (2017). Biology and clinical significance of circulating tumor cell subpopulations in lung cancer. Transl. Lung Cancer Res..

[35] Lindsay C.R., Blackhall F.H., Carmel A., Fernandez-Gutierrez F., Gazzaniga P., Groen H.J.M., Hiltermann T.J.N., Krebs M.G., Loges S., Lopez-Lopez R. (2019). EPAC-lung: pooled analysis of circulating tumour cells in advanced non-small cell lung cancer. Eur. J. Cancer.

[36] Tibbe A.G., Miller M.C., Terstappen L.W. (2007). Statistical considerations for enumeration of circulating tumor cells. Cytometry A.

[37] Martin M., Garcia-Saenz J.A., Maestro De las Casas M.L., Vidaurreta M., Puente J., Veganzones S., Rodriguez-Lajusticia L., De la Orden V., Oliva B., De la Torre J.C. (2009). Circulating tumor cells in metastatic breast cancer: timing of blood extraction for analysis. Anticancer Res..

[38] Allard W.J., Matera J., Miller M.C., Repollet M., Connelly M.C., Rao C., Tibbe A.G., Uhr J.W., Terstappen L.W. (2004). Tumor cells circulate in the peripheral blood of all major carcinomas but not in healthy subjects or patients with nonmalignant diseases. Clin. Cancer Res..

