# Supplementary material for: Pre-Analytical and Analytical Variables of Label-Independent Enrichment and Automated Detection of Circulating Tumor Cells in Cancer Patients
Source: Cancers (Basel). 2020 Feb 13;12(2):442. doi: 10.3390/cancers12020442 (PMC7072175; doi:10.3390/cancers12020442)
Supplement: Supplementary file 1 [file cancers-12-00442-s001.zip › cancers-701309-suppl-XML/cancers-701309- Figures S1-2 and Table S5.pdf]

Supplementary Materials: Pre-Analytical and Analytical Variables of Label-Independent Enrichment and Automated Detection of Circulating Tumor Cells in Cancer Patients

Claudia Koch, Simon A. Joosse, Svenja Schneegans, Okka J. W. Wilken, Melanie Janning, Desiree Loreth, Volkmar Müller, Katharina Prieske, Malgorzata Banys-Paluchowski, Ludwig J. Horst, Sonja Loges, Sven Peine, Harriet Wikman, Tobias M. Gorges and Klaus Pantel

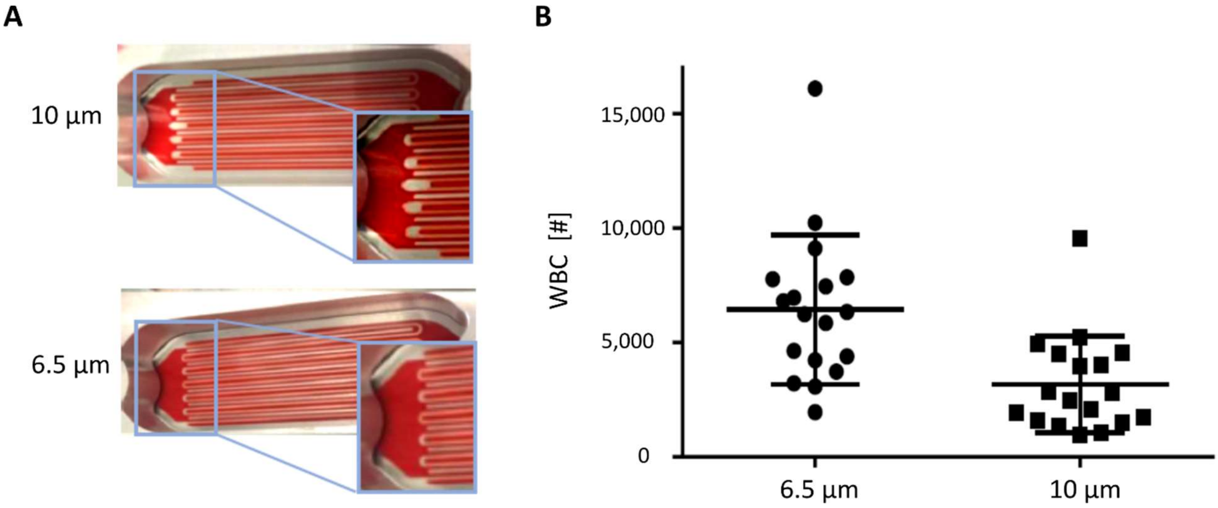

Figure S1. Cassette priming and white blood cell (WBC) count. (**A**) Representative pictures taken of sample processing following successful Parsortix® cassette priming for the 10 µm and 6.5 µm gap sized cassette. Blue boxes contain magnified picture elements. Prior to running blood samples, Parsortix® cassettes are primed with 100% ethanol and 1× PBS to ensure minimal air bubbles caught in the cassettes. The latter represent a hindrance for ideal sample processing. The 6.5 µm gap sized cassettes showed improved priming with no remaining air bubbles. (**B**) White blood cell (WBC) background increases from a median of approx. 3000 to 6000 remaining WBCs when decreasing the cassette gap from 10 µm to 6.5 µm in healthy donor blood samples drawn into EDTA.

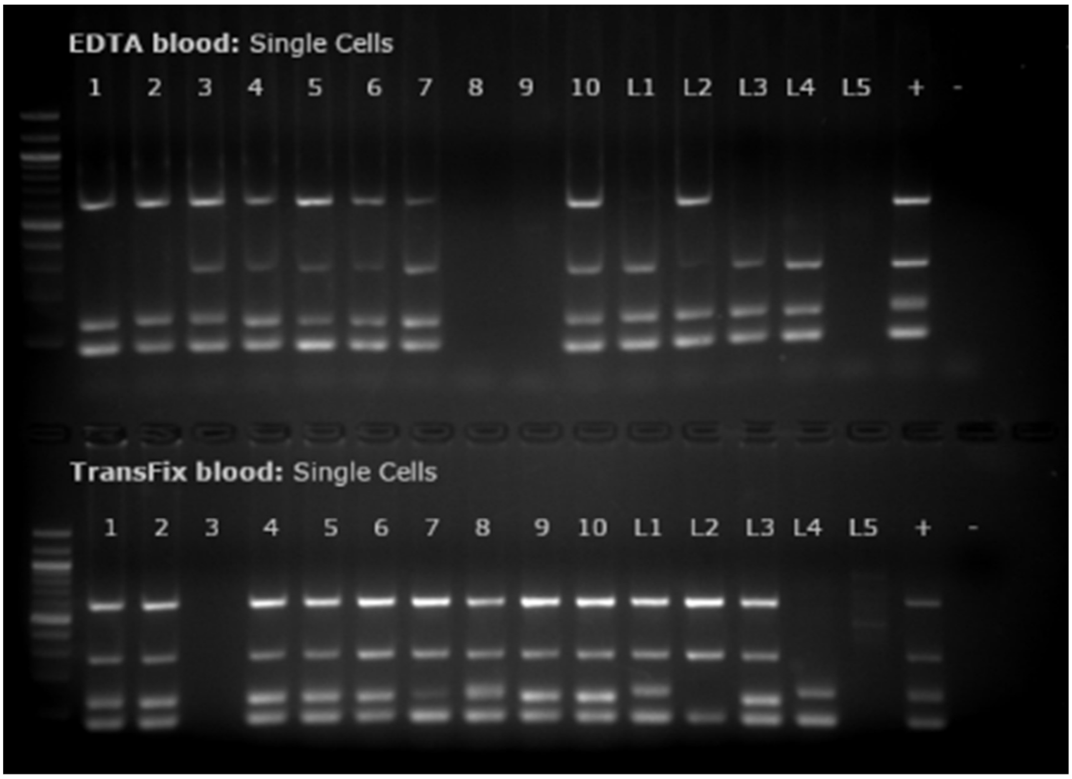

Figure S2. Efficiency of whole genome amplification (WGA) and results of DNA quality control on single cells processed with our optimized protocols. MDA-MB-468 cells were spiked into EDTA or TransFix® blood and processed via Parsortix® (6.5 μm cassette, 99 mbar). Subsequently, cells were stained, manually picked and DNA amplified using the Ampli1™ WGA kit (Menarini Silicon Biosystems). 10 single tumor cell line cells (1–10) and 5 leukocytes (L1–L5) were processed per blood tube type. gDNA was applied as positive (+) and H_2O as negative (−) control. Following amplification, DNA was subjected to quality control using the Ampli1™ QC Kit (Menarini Silicon Biosystems) and visualized via agarose gel electrophoresis.

Table 5. Detailed recovery results for 13 clinical cancer patient samples drawn in parallel into different blood tubes and processed with the 6.5 µm cassette gap via the Parsortix™ system. Clinical samples were obtained from metastatic non-small-cell lung cancer (mNSCLC), small-cell lung cancer (SCLC) and metastatic gastrointestinal cancer (mGIC) patients. In cases in which CTCs were found, results are presented as (x/y), x = total number of single CTCs, y = total number of CTC clusters detected.

Sample	Entity	EDTA (99 mbar)	EDTA (50 mbar)	CellSave	Streck	Transfix
UKE_1	mNSCLC	0	0	0	0	0
UKE_2	mNSCLC	0	0	0	0	0
UKE_3	mNSCLC	0	0	0	0	0
UKE_4	mNSCLC	0	0	0	0	0
UKE_5	mNSCLC	0	0	0	1	1
UKE_6	mNSCLC	0	1	4	2/1	4
UKE_7	mSCLC	0/1	3	2	4	9/1
UKE_8	mGIC	0	0	0	0	0
UKE_9	mGIC	0	0	0	0	0
UKE_10	mGIC	0	0	0	1	0
UKE_11	mGIC	0	1	1	0	1
UKE_12	mGIC	0	0	0	2	3
UKE_13	mGIC	1/1	2/4	2	2	3/2
≥1 CTC positivity rate [%]		15.4	30.8	30.8	46.2	46.2
Total amount of single CTCs		1	7	9	10	21
Total amount of clusters		2	4	0	1	3

© 2020 by the authors. Licensee MDPI, Basel, Switzerland. This article is an open access article distributed under the terms and conditions of the Creative Commons Attribution (CC BY) license (http://creativecommons.org/licenses/by/4.0/).
